# Using a Situation Awareness Approach to Identify Differences in the Performance Profiles of the World’s Top Two Squash Players and Their Opponents

**DOI:** 10.3389/fpsyg.2019.01036

**Published:** 2019-05-14

**Authors:** Stafford Murray, Nic James, Janez Perš, Rok Mandeljc, Goran Vučković

**Affiliations:** ^1^High Performance Sport New Zealand, Millennium Institute of Sport and Health, Auckland, New Zealand; ^2^Faculty of Science and Technology, London Sport Institute, Middlesex University, London, United Kingdom; ^3^Faculty of Electrical Engineering, University of Ljubljana, Ljubljana, Slovenia; ^4^Faculty of Sport, University of Ljubljana, Ljubljana, Slovenia

**Keywords:** situation awareness, performance profiles, squash shots, movement parameters, tactics

## Abstract

**Purpose:**

The pressure exerted on a squash player is a consequence of the quality of a shot coupled with the ability of the player to return the ball, namely, the coupling of the two players’ situation awareness (SA) abilities. SA refers to an awareness of all relevant sources of information, the ability to synthesize this information using domain knowledge and the ability to physically respond to a situation.

**Methods:**

Matches involving the two best players in the world (*n* = 9) at the 2011 Rowe British Grand Prix, held in Manchester, United Kingdom were recorded and processed using Tracker software. Shot type, ball location, players’ positions on court and movement parameters between the time an opponent played a shot prior to the player’s shot to the time of the opponent’s following shot were captured 25 times per second. All shots (excluding serves and rally ending shots) produced five main SA clusters, similar to those presented by Murray et al. (2018), except a greater proportion of shots were categorized in the greater pressure clusters and less in the lower pressure ones.

**Results:**

Individual matches were presented using cluster performance profile infographics which demonstrated how individual player’s performance profiles differed between matches.

**Conclusion:**

It is suggested that it is the coupling, of the two player’s behaviors, that makes the examination of tactics so challenging. This inherently means that performance profiles vary in subtle ways, making consistent profiles that are independent of the opponent very unlikely for elite players. This approach should be further modified to determine within match changes in performance.

## Introduction

In squash, like all racket sports, the main objective of any shot is to minimize the amount of time available to the opponent to hit their shot. This is optimally achieved by hitting the shot accurately and early, e.g., a volley, forcing the opponent to move quickly over a maximal distance. To counter this pressure, expert players can anticipate where the ball will go (Abernethy, 1990; Triolet et al., 2013) using a split step to initiate movement (James and Bradley, 2004) move efficiently on a well-defined path before lunging to hit the ball. This action also allows a very efficient return to the T area of the court, where winning players have been shown to spend a greater proportion of total playing duration than losers (Vučković et al., 2009). This means that two factors determine the amount of pressure exerted on a player: (1) the quality of a shot, and (2) the ability of the player to move to return the ball which involves knowing where the ball will go as soon as possible, potentially some anticipatory behavior. Triolet et al. (2013) estimated that elite tennis players demonstrated anticipation behaviors only between 6.14 and 13.42% of the situations analyzed, suggesting that, in most situations, tennis players do not need to exceed in anticipation actions, since sufficient ball flight information will enable them to return the ball without any risk. James and Bradley (2004) also found limited use of anticipation in expert squash players as they initiated their first movement toward the ball on average 270 ms (± 0.09 s) after ball contact, assuming a reaction time of approximately 200 ms, this suggested they often utilized ball flight information before moving. However, only relatively easy shots were sampled, to prevent situational probabilities from being used, suggested as a potential confounding variable by Abernethy et al. (2001). Whilst these studies suggested that anticipatory behaviors were not as prevalent as perhaps assumed, it is also possible that players could anticipate but chose not to. This could be because overuse of anticipation could be detected by their opponent and over anticipating could end up counterproductive, or anticipatory behavior simply enables the response to be planned and executed more effectively, often without the need for either an early movement or unnecessary speed.

A fundamental question, albeit difficult to answer, relates to which shot should be played in any situation. Whilst coaches often consider one shot optimal, usually when a player is under some pressure, it would be likely that expert players would usually select this shot. This would mean that discernible patterns of play, i.e., consistent shots played in certain situations, would be evident. Sanderson and Way (1977) tested a hypothesis related to this, i.e., that “an individual exhibits a pattern of play which is relatively stable over time and independent of the opponent.” Their results suggested that players showed a higher degree of similarity when winning compared to losing. The concept of a “pattern of play,” meaning the relative frequency of each stroke a player made in the matches analyzed, suggests that if players demonstrate a relatively stable playing pattern then opponents can make use of this information to their advantage. However, McGarry and Franks (1996) found that invariant (consistent) patterns of play were difficult to ascertain but suggested that the complexity of discriminating the situation in which the shot was played was a crucial factor. They suggested that the preceding shot alone was unlikely to be sufficient to predict the subsequent shot. In response, Vučković et al. (2014) controlled for previous shot type, time between shots, court location and the handedness of the players. They found that tight shots (played from close to the corners of the court) tending to be more predictable (two or three typical shots played) compared to loose ones (up to seven different shot responses to the same preceding shot when nearer the middle of the court).

Murray et al. (2018) described shot selection in squash from a situation awareness (SA) perspective (Endsley, 1995). SA refers to the awareness of relevant sources of information, the synthesis of this information using domain knowledge gained from past experiences (Abernethy et al., 2001) and the ability to physically respond to the situation. Murray et al. (2018) suggested the relevant sources of information were likely to be related to events previously encountered (historical and within the game being played), opponent movements (visual cues) and probabilistic information such as a heuristic “in this situation it is likely that…...” This perspective demonstrates the complexity in deciding which shot to play and raises the question as to what extent individual differences affect this decision-making process. Within this SA perspective the final task of actually playing the shot is important since an inaccurate shot would give the opponent a relatively easy shot under no time pressure and thus offset any advantage gained from having successfully accomplished the first two tasks, e.g., identified the opponent’s shot early and been able to volley the ball and hence reduce time.

Previous research has tended to analyze relatively large data sets, grouping individual players according to their level of expertise, e.g., Vučković et al., 2014; and may be inappropriate, e.g., grouping attacking players with defensive ones. This aproach fails to consider individual differences, potentially falling into what Mackenzie and Cushion (2013) identified as a “theory-practice gap,” where research findings were suggested to have a lack of transferability and had little or no relevance to practitioners in sport. They advocated that performance analysis research should be for practitioners to utilize the results to improve performance. To address this issue, more discriminating information relating to, processes rather than just outcome measures (James, 2009), and in relation to individual, rather than multiple, players or teams are required.

Murray et al. (2018) presented six shot type clusters, referred to as SA clusters, named to relate to the outcome of a shot ranging from a “defensive” shot played under pressure to create time to an “attempted winner” played under no pressure with the opponent out of position. The important point was these authors used the term SA to reflect the point that the clusters represented both the intention to play a specific shot, based on the situation the player was in, and the outcome of the shot in terms of the effect the shot had on the opponent’s movement. They used a two-step cluster analysis using two distance parameters (how far the player moved to return the shot and the distance the player was from the T at the moment the shot was hit) as well as the time and maximum velocity of the player returning the shot (between the shot and the returning shot). They only used shots that were played from selected areas of the court (front, middle, or back) that had achieved their objective, namely the ball was returned from the area of the court aimed for. The logic for this decision being that shots that did not achieve their objective would have, potentially significant, different movement parameters, e.g., when an opponent anticipated a shot and was able to volley the ball or the shot was played badly enough to allow this type of interception. By only analyzing shots that achieved their objective, the authors were able to differentiate different SA clusters for the same shot type from the same court area, suggested as being consistent with players changing the pace and trajectory of the shot because of different objectives (SA tasks). However, this selection process removed around 50% of shots from the analysis, the less accurate shots, and therefore presented a distorted view of overall shot outcomes and an inaccurate evaluation of players’ performance. Therefore, this study aimed at presenting a more accurate picture of shot distributions in elite male squash players and also increasing the likelihood of finding between player differences in shot outcomes, since shots that achieved their objective impacted their opponents similarly. The present study had a further purpose to increase the ecological validity of a previous study (Murray et al., 2018), using all shots irrespective of their outcome.

Previous papers have grouped players according to their world ranking (e.g., Hughes and Robertson, 1998; Murray et al., 2016) but we argue that players are always moving up or down the ranking list and their current world ranking may not be an accurate reflection of their ability at the time a match is played. This is particularly obvious for young emerging players or older players moving down the ranking list. Similarly, players may have different strengths and weaknesses meaning that they play with somewhat different approaches, e.g., high tempo risky versus defensive attrition. Grouping these different players together will therefore reduce the accuracy of the analysis. It is the aim of this paper, therefore, to also compare the shot selections, and shot effectiveness, of two elite players, ranked as the top two players in the world at the time of data collection, using shots that both achieved and did not achieve their objective, i.e., where the return shot was played from was not a factor check except for lobs which were returned from the front of the court as this very unusual situation was removed from the analysis. This approach will provide a more detailed analysis of the differences evident between players of very similar ability and provide more practically relevant information.

The methodology used in this paper led to a couple of hypotheses. First we thought that individual players would exhibit different playing patterns between matches, due to not playing at full ability against weaker opponents, rendering grouping players, and matches as meaningless in terms of practical significance. Secondly, we hypothesized that different playing styles would be apparent if an in-depth analysis of shot types was included. Squash pundits and fans consider the game has changed with a more attacking style favored by some, in particular the Egyptians who currently dominate the sport. Our analysis of the World number 1, an Egyptian, was thus thought to be likely to provide evidence of this attacking style of play.

## Materials and Methods

### Participants

Matches at the 2011 (*n* = 9) Rowe British Grand Prix, held in Manchester, United Kingdom were recorded and processed using Tracker software (Vučković et al., 2014), a newer version of the SAGIT/Squash software (Perš et al., 2008). Ten full-time professional players (age 28.8 years ± 2.95 years), who were ranked in the world’s top 64, participated in this study. The Professional Squash Association granted approval for all data capture and analysis of their players for research purposes and ethical approval for the study was provided by the sports science sub-committee of Middlesex University’s ethics committee in accordance with the 1964 Helsinki declaration.

### Data Collection and Processing

Matches took place on a court set up with a PAL video camera (Sony HDV handy camera HVR-S270, Japan) with a specially adapted 16 mm wide angled lens (Sony NEX SEL16F28) attached to the ceiling above the central part of the court such that the entire floor and part of the walls were within the field of view. A similar camera (used by the Professional Squash Association to record matches) was located on a tripod 15 m behind the court and 5 m above ground level. The camera placement and techniques for transferring video images into Tracker were identical to SAGIT/Squash, i.e., automatic processing with operator supervision, and have been well documented (Vučković et al., 2009). Similarly, the reliability for resultant calculations of distance and speed for each player (Vučković et al., 2010) and positions on court (Vučković et al., 2009) have been published. The exact camera location for the overhead camera (both vertically and horizontally) was not critically important, as subsequent calibration for image capture accounted for its position. Data were collected 25 times per second.

**FIGURE 1:**
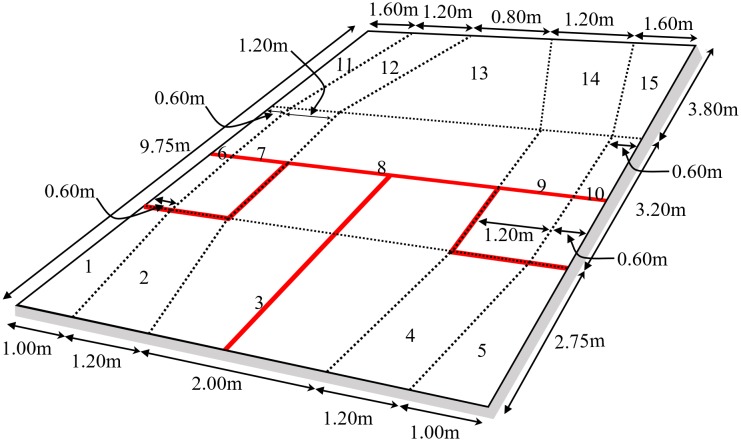
Squash court floor divided into 15 cells.

The shot type (*n* = 24; Table 1) and ball location (cell, Figure 1) for each shot (denoted player A), excluding serve, return of serve, and rally ending shots (winners, errors, lets and strokes), were recorded along with the same information for both the preceeding shot (B-1) and following shot (B+1). The justification of the cell dimensions was originally presented by Vučković et al. (2014) who suggested that shots near the sides of the court were far more critical than central areas, arguing that the area of the cells should reflect this. They also noted that the ball bounced differently when it hit the sidewall and using this sidewall bounce was a deliberate tactic in elite squash. Whilst this is tactically astute the authors pointed out that the resultant trajectory of the ball tended to finish further away from the sidewall the nearer the ball got to the back wall. A similar observation was made at the front of the court. On this basis the authors argued that cells should not be rectangular in the front and back of the court but should represent typical ball trajectories for these areas. Once the basic shape of the court cells had been identified reliability studies were carried out to determine the optimum area of the cells. These tests resulted in the 15 cells used in this study with the acknowledgment that smaller cells would provide better distinction of shot difficulty but the consequent lowering of reliability meant that for this data collection method smaller cells were not possible.

**Table 1:** Operational definitions for shot types used.

Shot type	Variations	Number	Definition
Drive	Straight or crosscourt Groundstroke or volley Hits back wall or not	8	The most prevalent shot in squash is the drive which aims to push the opponent into one of the two back corners of the court. The shot can be hit at different speeds and heights on the front wall primarily determined by the tactical situation.
Boast	Two or three wall Groundstroke or volley	4	The shot is hit onto the side wall prior to the front wall. The objective is to move the opponent into one of the two front corners of the court. The basic two wall boast aims to force the opponent to hit the ball before the ball reaches the opposite side wall. The three wall boast can be aimed for the opposite wall nick (join between wall and floor) and if played well can be a winning shot but the three wall boast can also be played as a very defensive high shot.
Drop	Straight or crosscourt Groundstroke or volley	4	A low soft shot to move the opponent into one of the two front corners of the court. The side wall is usually a secondary target to increase opponent difficulty.
Kill	Straight or crosscourt Groundstroke or volley	4	A low hard shot to move the opponent into one of the two front corners of the court. Hitting hard gives the opponent less time but the side wall has to contribute to the opponent’s difficulty otherwise the shot can be poor.
Lob	Straight or crosscourt Groundstroke or volley	4	A high soft shot to move the opponent into one of the two back corners of the court. The main objective is to enable the player to recover the T area before the opponent plays a shot.

Additional information regarding time, speed and distance were recorded both between shots and at the time player A hit the ball (see Murray et al., 2018 for original methods who explained how the original list of variables was reduced incrementally by removing the least powerful predictor from an analysis due to poor clusters being formed. This was repeated until clusters deemed fair were found). The resultant information used in this paper was thus both following player A’s shot, i.e., variables related to player B’s movement, and considered as measures of the shot’s effectiveness. Other information both prior to the shot and at the time of the shot, the opponent’s position relative to the T area was used in this paper, which may have reflected the player’s SA (Macquet, 2009) and hence influenced decision-making. This study did not differentiate the same shot when played from different areas of the court as did Murray et al. (2018), rather shots were classified by type, e.g., straight drive, irrespective of whether it was played from the front or back of the court. This procedure was considered more appropriate since all rally continuing shots were analyzed, rather than only the ones that achieved their objective, as Murray et al. (2018) did. This meant that the variability associated with the variables collected was far greater and this complexity prompted the simplification of the shot classification. One shot was removed from the data (lob from front of the court that was volleyed in the front of the court) as the variables collected suggested this was an attacking shot. This was, however, either a poorly executed defensive shot or a very unusual interceptive movement by the opponent. For elite players, both situations are rare and were hence deemed outliers and removed.

### Statistical Analysis

Each shot in squash has an objective, which in simple terms, is to place the opponent under as much pressure as possible given the constraints of the situation. This ranges from applying a lot of pressure when in a good situation to minimizing an opponent’s advantage when in a poor situation. Coaches may not always agree on what the objective was, or should be, in every situation, e.g., did a player try to play a winning shot or just apply pressure on an opponent? This would also be determined by the execution of the shot as a well-played shot would have different consequences to a less accurate one. The same cluster analysis of a previous study (Murray et al., 2018) was used for this study. This is a data mining technique that enables the formation of groups within a data set based on maximizing the homogeneity of cases within a group and the heterogeneity between clusters (Hair et al., 1995). Cluster analysis begins with all cases as separate groups and the two “most alike” cases are combined in the first step using the most appropriate distance measure. The two cases with the smallest distance measure will then cluster together and a group mean (cluster centroid) can be calculated and used in the next step. The next two most alike cases (or groups once cases have been clustered) are then combined. This process continues until an optimal cluster solution is obtained, although this may be determined from a practical standpoint as there are no objective methods for determining the optimal number of clusters (Hair et al., 1995).

The two-step cluster analysis, using a probability-based log-likelihood distance measure (SPSS) enabled the same continuous (two distance parameters, time, and maximum velocity) and categorical (shot type) variables to be used in a single analysis. However, when running a cluster analysis on different data, we used all shots rather than Murray et al.’s constrained shots, different clusters were found from those reported by Murray et al. (2018). The cluster parameters in this study, i.e., all players, all shots, were very similar, however, hence we used the same names for the new clusters. The silhouette coefficient, i.e., the measure of cohesion and separation for clusters, was lower (average = 0.2) compared to the 0.35 found in Murray et al. (2018). The importance of each continuous predictor variable was 1.0 with the exception of opponent distance to T which was 0.85. Differences became more marked, however, when individual players were analyzed, necessitating the need to quantify which original cluster each new cluster was most similar to.

#### Determining Which Was the Most Similar Cluster

Each cluster was determined by the group mean (cluster centroid) based on the four continuous (two distance parameters, time, and maximum velocity) and one categorical (shot type) variable. To determine which cluster (all players, all shots) each individual player cluster most resembled, the absolute differences, between the means for each continuous variable for one individual player cluster and the same variable for all clusters (all players, all shots) were calculated. The cluster which had the lowest sum, of the four absolute differences, was hence deemed the most similar. On this basis, an individual player’s clusters were color coded according to the colors of the most similar clusters used for the general, all players, all shots, clusters. Hence, an individual player cluster profile did not always exhibit the same five clusters as for the general profile meaning that different color profiles were often generated.

#### Determining the Degree of Difference Between the Clusters

Having determined which general cluster each individual player cluster was most similar too, and hence color coded the same, the degree of difference between the two clusters was calculated as an additional check that the color coding was appropriate. This was achieved by finding how far the mean for each parameter, for the individual player, was from the mean of each parameter, for all players, in terms of standard deviations, i.e., the z score. The four z scores were then summed, not averaged because scores could be both negative and positive, to give an overall deviation value. The maximum z scores obtained from all clusters presented in this paper were ± 0.92.

## Results

**FIGURE 2:**
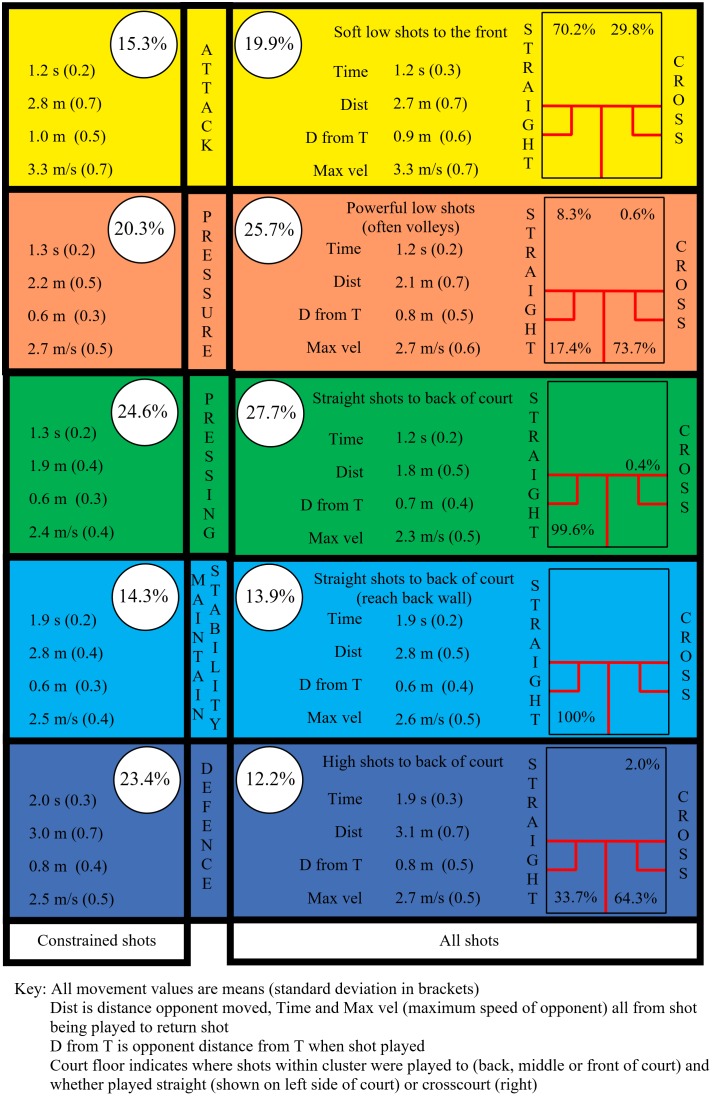
Time, distance, and speed parameters for five SA clusters using constrained shots (Murray et al., 2018) compared to all shots (all data from Murray et al., 2018).

Five SA clusters were named, the same as for Murray et al.’s (2018) constrained shot approach, to relate to the outcome of a shot (Figure 2). When all shots from Murray et al.’s (2018) data set were used, the proportion of shots creating the most pressure on the opponent, increased in comparison to the previously used, constrained shot approach. This was primarily due to there being 11.2% less defensive shots and a corresponding increase in offensive shots (4.6% more attack, 5.4% more pressure, and 3.1% pressing). The parameters for each cluster remained very similar, however, with the biggest difference being for maximum velocity in the defense cluster (increase of 0.2 m/s). General descriptors that described the shot types associated with each cluster were added to Figure 2, e.g., attacking clusters shots tended to be soft shots to the front, although occasionally a very small proportion of a different shot type was associated with a cluster, i.e., crosscourt shots (0.4% of pressing) and three wall boast (2.0% of defense). Shots in the attacking cluster aim to increase the distance and reduce the time for the opponent, hence the highest maximum velocity of any cluster seen for the opponent. Shots tended to be played straight to the front (70.2%) rather than crosscourt. In comparison, the pressure cluster showed how elite players can use different shots, played to all four corners of the court, to exert similar levels of pressure on the opponent.

**FIGURE 3:**
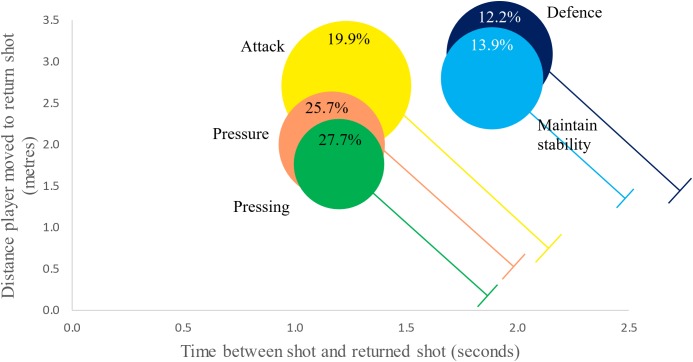
Shot clusters for all shots (all data from Murray et al., 2018).

In order to present the different clusters relative to each other, whilst also presenting all four variables, a cluster performance profile infographic was created (Figure 3). The center of each circle (cluster) is located according to the mean value for time (x) and distance (y), between the shot being played and the return shot. The distance the opponent was from the T at the time of the shot is represented by the length of the T which is drawn relative to the *x* axis. Finally, the diameter of the circle is proportional to the maximum speed the opponent ran to return the shot. This infographic depicts three attacking (attack, pressure, and pressing) and two defensive (defense and maintain stability) clusters.

**FIGURE 4:**
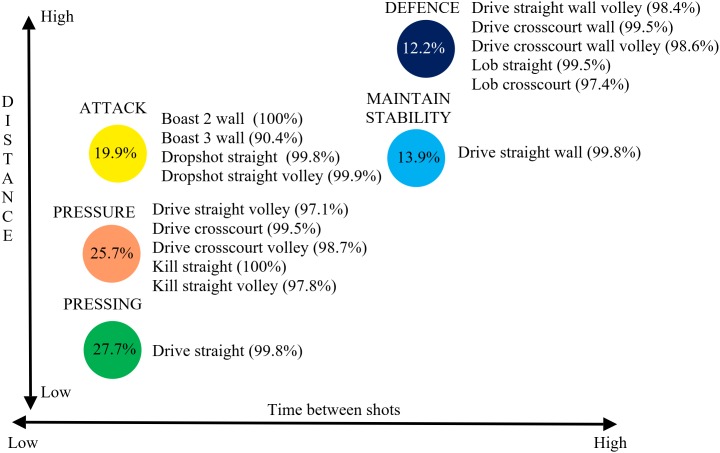
Shot types categorized into different clusters for all shots (all data from Murray et al., 2018).

The infographic (Figure 3) was then used for all nine matches involving the World number 1 and 2 players (their performances in the middle and their opponents outside) culminating in the final played between them (Figure 4). Each match demonstrated different cluster patterns (performance profiles) with matches involving the World number 1 displaying a tendency for greater pressure to be exerted as the standard of the opponent increased, 30.9% defensive shots (Ashour, 38% his opponent) against the opponent ranked outside the World’s top 24 compared to 22.1% (24.7% opponent) against his top 8 ranked opponent.

**FIGURE 5:**
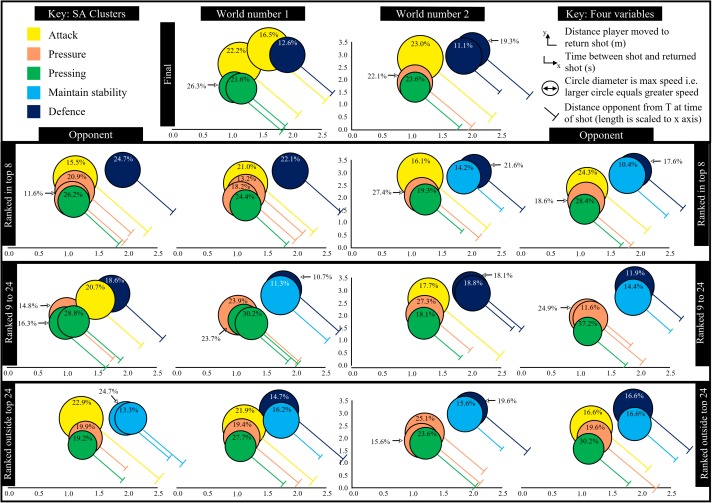
Shots clusters for matches involving Ramy Ashour (World ranked number 1) and Nick Matthew (World ranked number 2).

**FIGURE 6:**
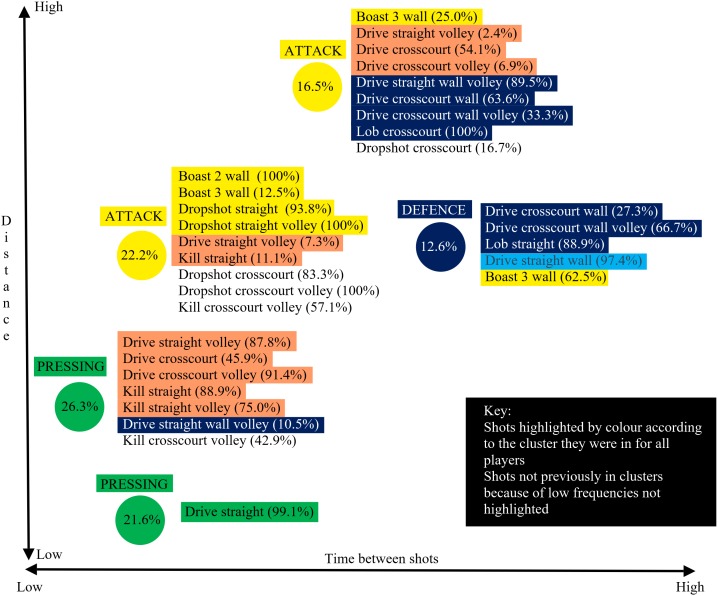
Shot types categorized into different clusters for Ramy Ashour (World ranked number 1) when playing against Nick Matthew (World ranked number 2).

In the final (Figure 5) the World number 2 forced his opponent to move slightly further with more accurate shots to the back (volleys straight, crosscourt drives) and front of the court (straight kills, volley straight kills) categorized in the pressure cluster whereas these shots were categorized in the pressing cluster for the World number 1. In contrast, the World number 1 gave his opponent less time on shots usually associated with the defense cluster (as they were for world number 2). Hence, 54% of his crosscourt drives that reached the back wall, 89.5% of volleys straight that reached the back wall, 37.5% of 3 wall boasts and 100% of crosscourt lobs were categorized in an attacking cluster. To illustrate the extent to which players can hit shots that achieve different levels of pressure for the opponent an in-depth analysis of the World number 1’s shots for the final against the World number 2 is presented (Figure 6).

## Discussion

Traditional analysis of the tactical behavior of racket sport players has usually assessed the different shots played in different areas of the court. However, this approach has tended to fail to differentiate the small differences between individual elite players and to obtain practically valid differences we considered that a more in-depth analysis was needed. This paper focused on the amount of pressure exerted on an opponent by each individual shot and measured by three movement and one time variable. However, the amount of pressure exerted on a squash player is a consequence of the quality of a shot coupled with the ability of the player to move to return the ball. The categorisation of shot types according to four variables associated with opponent movement therefore encapsulates both the quality of the shot and the opponent’s ability to offset the pressure. Murray et al. (2018) focussed more on the former part of this pressure, namely the pressure exerted by the shot, as they only selected shots that achieved their objective. They removed shots where the opponent volleyed the ball in the middle of the court for example, often a consequence of anticipating the ball trajectory. This approach was deemed to discriminate decision-making where the same shot type played from the same court area produced different outcomes (SA clusters) as this was suggested as consistent with players changing the pace and trajectory of the shot because of different objectives (SA tasks).

This study adopted an alternative approach and included shots that did not achieve their objective, in other words shots which were played less accurately or where the opponent was able to anticipate and return the ball early. This approach complicates the analysis as more factors are likely to determine the amount of pressure a player is under but clearly has greater ecological validity in that this is a more accurate reflection of elite squash match play. Murray et al. (2018) named clusters with terms that were representative of the increasing pressure being placed on an opponent. This increased pressure was exhibited by the reduction of time available, differentiating the two defensive clusters and the defensive clusters from the attacking ones, and the increase in speed required of the opponent differentiating the attacking clusters. This quantitative approach derived clusters by the values of the parameters but the cluster names were derived from the squash expertise of the authors who used labels to reflect the aim of the shots (see Figure 4 for shot types used in each cluster). Utilizing the approach of using all shots (excluding rally ending shots as these require a separate analysis; see also Murray et al., 2018) five main SA clusters were found to be very similar to those presented by Murray et al. (2018). The attempt winner cluster only accounted for 0.6% of shots in this study and was thus not presented (Figure 2). The clear impact of using all shots was that a greater proportion of shots were categorized in the greater pressure (advantage situation) clusters (pressing, pressure, and attack) and less in the lower pressure (disadvantage or neutral situation) ones (maintain stability and defense). This gives a more realistic view, than Murray et al.’s (2018), of the amount of pressure elite male players tend to be under in match play conditions. In Figure 2 schematics of the court floor were included to highlight the different types of shot used within each cluster even though the parameters were similar. For example, the attack and pressure clusters exhibited similar values for the parameters but the placement of shots showed quite different approaches, with different shots achieving similar pressure on the opponent. The shots in the attack cluster were to the front of the court, hence less distance for the ball to travel (less time), also the ball tends to stay very tight to the sidewall (more difficult for opponent) for straight shots compared to crosscourt where the ball can easily move toward the center of the court if not played very well.

The relationship between the three movement and one time variables that defined each SA cluster was not clearly presented by Murray et al. (2018) prompting the creation of an infographic in this paper. The challenge of presenting four dimensions was alleviated by using just two dimensions (time and distance) with the other two represented by the size of the circle and length of T. This clearly differentiated two low and three high pressure clusters when all players and all shots were used. However, this overview of multiple players lacks the transferability in relation to individual players, the so called “theory-practice gap” (Mackenzie and Cushion, 2013).

Individual matches were presented to highlight how individual players exhibited different cluster formations in different matches. A fine-grained analysis of the final, played between the two top players in the World at the time, exposed some of the subtle differences, of relevance to practice (Mackenzie and Cushion, 2013), due to spatial and temporal variations within rallies. For example, the World number 1 gave his opponent less time on crosscourt drives and volleys straight (shots categorized in attack clusters, Figure 4), including shots which reached the back wall and usually associated with the defense cluster. This can be due to hitting these shots harder, hence reducing the time available. This would predominately be a consequence of the quality of a shot, since the opponent was unable to return the ball early, but other factors, such as opponent positioning, could be contributory. This typically occurs when an opponent moves forward to cover a short shot and is thus slightly out of position for a shot played to the back of the court. This in-depth analysis also showed his overall capability of utilizing more offensive tactics. He used a larger array of attacking shots (Figure 6, shots not highlighted in the attack clusters) and was able to exert more pressure for some drive straight volley (7.3%) and kill straight (11.1%) shots at a level consistent with the attack cluster. This was also evident for all shots previously (Figure 4) associated with the pressure cluster (highlighted orange in Figure 6), but in this match the world number 1’s shots produced values more closely associated with attack and pressing clusters. This in-depth analysis clearly identified strategy changes for individual players between matches (Figure 5) but also how individual shot types can have different outcomes within a match (Figure 6). Whilst Figure 6 showed the general relationship between each cluster the figure was not scaled perfectly due to the main aim of showing which shots contributed to a cluster. The differences between Figure 4 (all players multiple matches) and Figure 6 (World number 1 playing a match against World number 2) demonstrated how a general picture derived from a large data set does not accurately portray the individual differences evident between players or even between matches. One clear advantage of the in-depth match analysis (Figure 6) was evident in the fact that the Boast 3 wall was seen in both attack and defense clusters. People knowledgeable in Squash would not be surprised at this as the shot can be played in two polar opposite ways but was only classified as an attacking shot in the general model because of the relatively infrequent use of the shot in defense.

This paper sought to present useful information at the practice level through an in-depth analysis of the world number 1 player in one match, but also sought to present evidence that players do not play the same way againset all players, Figure 5, by showing how individual match clusters differed from a general picture of elite male squash. Whilst focusing on the World number 1 in this regard, it was clear that he tended to increase the pressure on opponents as the opponent quality increased. This is suggestive of a strategy of playing within himself when the opponent threat was minimal but when necessary his performance levels increased. This supports the finding of McGarry and Franks (1996) who found consistent patterns of play elusive. However, their work comprised a sample of 8 elite players taken from 10 matches where invariant patterns of play would be less likely than for one player in one match as presented here. The degree of difference, both between and within players, found here suggests that many researchers have previously underestimated the extent that individual differences play in decision-making processes, in this case deciding which shot to play. Equally, the complex coupling of the two players (shots and movements) can lead to differences in shot outcomes, e.g., defensive type shots can result in high pressure for the opponent (classified as an attacking cluster) because of spatial and temporal effects present during the rally. This type of effect can be as a consequence of very small differences in the movement or positioning of a player which, for example, prevents the usual volley return and forces the less advantageous ground stroke response.

Motion and time information was used to elicit small differences between and within players, evident between matches. These methods are applicable for other racket sports and have been used in tennis (Kovalchik and Reid, 2018). Further advances are likely as researchers become more adept at using computer science methods to discern meaningful patterns in complex data sets like these.

## Conclusion

This paper has further demonstrated the usefulness of analyzing squash from a SA approach but has also demonstrated the inherent variability associated with squash match play. The dynamic between the player trying to put pressure on an opponent by playing accurate shots is offset to some extent by an opponent who move efficiently thanks to an awareness of relevant sources of information and the synthesis of this information using domain knowledge gained from past experiences (Abernethy et al., 2001). It is this coupling, of the two player’s behaviors, that makes the examination of tactics so challenging. However, this is exacerbated because player’s decision-making abilities are unlikely to be the same between players, can change as a consequence of experience (even within a match) and may be incorrect on occasion. Whilst this approach has identified some of these complexities, highlighting within player differences between matches, within match changes in performance have still not been addressed. Until analysis procedures are sensitive enough to discern these differences it is unlikely that a true understanding of expert performance will be forthcoming.

## Ethics Statement

Ethics approval for the study was provided by the sports science sub-committee of Middlesex University’s ethics committee in accordance with the 1964 Helsinki declaration.

## Author Contributions

JP and RM assisted in the data acquisition, processing, and software development. SM, NJ, and GV designed the study, conducted the analysis, interpreted the data, and wrote the manuscript. All authors read and approved the final manuscript.

## Conflict of Interest Statement

The authors declare that the research was conducted in the absence of any commercial or financial relationships that could be construed as a potential conflict of interest.
